# Global Health Trade Summit: an AI-enhanced simulation of international trade and global health for undergraduate public health education

**DOI:** 10.3389/fpubh.2025.1681199

**Published:** 2025-11-14

**Authors:** Uday Patil, Melissa Terada, Denise C. Nelson-Hurwitz

**Affiliations:** Department of Public Health Sciences, Thompson School of Social Work & Public Health, University of Hawaiʻi at Mānoa, Honolulu, HI, United States

**Keywords:** global health education, public health pedagogy, experiential learning, trade, undergraduate

## Abstract

**Introduction:**

The Global Health Trade Summit is an undergraduate classroom activity designed to help students understand the role of international trade in addressing population health needs, especially given recent news about trade disruptions, health inequities, and access challenges. This economic simulation incorporates experiential learning and a constructivist pedagogical style, best practices in undergraduate public health pedagogy.

**Methods:**

This card game activity, designed with the assistance of artificial intelligence tools OpenAI ChatGPT and Adobe Firefly, involved gathering historical news and economic data, resource allocation, outcome simulation, and image generation. It aligns with objectives outlined in the guidelines for public health education set forth by CEPH, ASPPH, and CUGH. Pilot testing of the activity was facilitated by an instructor and teaching assistant with 39 students in an introductory global health course.

**Results:**

Students fully participated and remained engaged throughout the pilot activity, which closely mirrored resource management and trading mechanics prevalent in popular board and card games. The pandemic context resonated with them, enhancing their comprehension of international trade and its associated macroeconomic concepts, such as international aid, supply chain disruptions, and resource distribution challenges. Students reported enjoying the activity and provided valuable suggestions for enhancing the simulation’s effectiveness.

**Discussion:**

The success of the pilot activity underscores the value of engaging economic games in public health instruction. These activities are facilitated or enhanced through the utilization of readily available artificial intelligence tools for education. Recommendations for other instructors are also provided, along with opportunities for research.

## 1 Introduction

### 1.1 Background

Undergraduate curricula rarely teach at the intersection of international trade and public health; yet, integrating these disciplines is essential for preparing students to navigate an increasingly interconnected world. First, a clear grasp of trade dynamics is vital for understanding how medical supplies, health technologies, and even pathogens traverse borders—shaping both the emergence of infectious diseases and the capacity to respond (1, 2). Second, trade agreements and policies directly influence social determinants of health, affecting access to essential medicines, availability of nutritious foods, and safety of environments through economic development and regulatory frameworks (2–6). Third, not all health science students can conceptualize resource allocation or the effect of trade on availability and distribution of vital health resources, which is essential for grasping current health inequities (3, 7). Equipping students with this interdisciplinary perspective enables them to critically assess policies and contribute to evidence-based solutions that are both life-saving and cost-effective. This integration is particularly important given the dominance of international trade and aid-related discourse in the news (8–10) and the widespread misunderstanding of what “trade” actually entails (11, 12). These are misconceptions that ultimately impact millions of lives. For these reasons, interdisciplinary activities that introduce students to these concepts are necessary.

The University of Hawai‘i at Mānoa’s Bachelor of Arts in Public Health (13) program has a well-established history of innovation through the creation of curricula and activities (14–19). Experiential learning is the hallmark of undergraduate education in this program, backed by nationwide best practices (20–22). After a period dominated by remote and hybrid teaching models due to the COVID-19 pandemic, there has been a return to in-person, group learning activities, with early indications of better academic outcomes in students at all education levels (23–28).

In line with calls to decolonize global health education in a post-pandemic era, the Introduction to Global Health course, offered by the Department of Public Health Sciences, has been adapted to center the voices, expertise, and agency of individuals from low- and middle-income sovereign states, emphasizing their roles in solving global health challenges alongside wealthier counterparts (29). Additionally, the course answers voices that advocate for participatory, critically reflective approaches that empower students to co-construct knowledge and deepen their understanding of global systems (30). The challenge for instructors is to create activities that incentivize students to engage with these complex topics in a meaningful way.

The advent and adoption of generative artificial intelligence tools, particularly large-language model-driven chatbots and image generation applications, are revolutionizing the way instructors create activities and even changing the purpose of education (31–33). These tools have made interdisciplinary simulations easy to create and package as classroom activities. Artificial intelligence (AI) is enabling instructors to create novel and personalized experiences not considered before.

Recently, instructors created and piloted an interdisciplinary, experiential, AI-driven activity, the Global Health Trade Summit, within the Introduction to Global Health course. This activity aims to transition students from viewing global health as a collection of regional-focused health topics to comprehending global health from a systems perspective, where economics, politics, and disaster management play a significant role.

### 1.2 Purpose and rationale

The Global Health Trade Summit was created and piloted to help students comprehend the significance and intricacies of international trade in addressing population health concerns. Students in the course expressed interest in better understanding international trade and connections to global health early in the semester. This focus is especially pertinent given Hawaiʻi’s tragic experiences of illness, with diseases introduced by traders and colonists (34), as well as recent news about trade disruptions and ongoing challenges to health equity and access (8–10). Teaching undergraduate students fundamental concepts of international trade can help them place their knowledge in a larger context through systems thinking (35) and provide “opportunities for students to find and develop a passion for issues that advance the human condition broadly, making for an active, articulate, and engaged citizenry” (36). Specifically, students discover how global supply chains affect the availability, cost, and equitable distribution of essential health resources. Through immersive role-play scenarios, students gain practical experiences that foster ongoing reflection and strategic thinking.

To streamline the development of this educational activity and ensure consistent, meaningful experiences for all students, artificial intelligence tools were intentionally integrated. These tools simplified the time-consuming and technically complex task of building a simulation, structuring the activity with clear mechanics that support learning objectives. In addition to supporting technical design, AI tools also facilitated the integration of interdisciplinary content—such as macroeconomic information from historical events from sovereign states around the world—and enabled the generation of custom imagery, resulting in a visually rich experience. The design combines elements of resource management and trade, common in economic simulations, with interactive gameplay features inspired by popular card and board games, making it both educational and engaging. Moreover, the pilot was developed to align with prominent global health education standards (37–39), ensuring that it will captivate students while upholding essential educational standards in many schools.

## 2 Pedagogy

### 2.1 Frameworks

This activity incorporates various pedagogical frameworks commonly employed in general undergraduate and public health studies. The constructivist pedagogical principles employed in this activity emphasized active learning, where students actively participate in creating meaning through activities (40–42). This approach is repeated in an experiential learning cycle, initially developed by Kolb and colleagues, encompassing four stages: experiencing, reflecting, conceptualizing, and experimenting (43).

Rules and mechanics underpinning this activity were drawn from economics and game theory, where gameplay of collectible card games and resource management games are often derived. To increase motivation and excitement to learn, the instructors incorporate gamification, defined early as “the use of game design elements in non-game contexts” (44, 45). Studies and reviews report that gamification, and the use of serious games, can raise student motivation and can improve learning outcomes in various levels of and disciplines within higher education (45–50).

The instructors designed each game element to encourage communication and reflection, skills the course was designed to train. The game elements included time-limited rounds, roles, resource tokens, surprise event cards, and quick feedback. Each game element was selected to align with a specific learning objective. Timed rounds engaged students in practicing decision-making under constraints; role assignments created conditions requiring collaboration and leadership; resource tokens effectively conveyed scarcity and trade-offs; and event cards required systems thinking, specifically in dealing with shocks and policy responses. These mechanics also supported ethical reasoning regarding equity, and facilitated progress toward outcomes evident in students’ negotiations, debriefs, and reflections.

Gamified or game-based learning environments draw from principle concept of gamification, and utilize game content and gameplay to enhance knowledge and skill acquisition (45–47, 51–53). Activities in these environments typically involve problem-solving spaces and challenges that provide players or learners with a sense of accomplishment (44, 48, 49, 51, 52). This differs from standard simulation in education. This activity was a gamified simulation, which incorporated game elements into a learning scenario. In contrast, a regular simulation simply models real events without these additional motivators (44, 45, 47, 53–55).

Undergraduate public health programs stress high-impact practices that build critical thinking, teamwork, and real-world problem solving through communication (38, 56). They also aim to prepare students for professional roles and for graduate study. This activity fits this approach by training students in those practices. Students take on an integrative task where they must weigh evidence, make policy choices, and explain trade-offs in plain language. These frameworks and guides ensured that strategic thinking and decision-making skills relevant to global health trade and policy were rooted within the activity.

### 2.2 Principles

The pilot activity is structured around four fundamental pedagogical principles.

First, students in the health sciences actively engage in role-playing scenarios (54, 57–60), making decisions that enhance their level of involvement and retention.

Second, the activity promotes collaborative learning (35, 61–64) through teamwork and communication, simulating real-world, allied health collaborations.

Third, iterative reflective practices (64–67) ground students in their own perspectives and provide individual time before collaborating with teammates. This fosters a deeper understanding of complex concepts and promotes continuous improvement.

Fourth, the activity was designed to develop ethical decision-making skills (68–71) by challenging students in scenarios of limited resources and many population health concerns. The activity encourages students to grapple with difficult choices, prompting reflection on the complexities of equitable resource distribution.

### 2.3 Competencies and standards

This pilot was designed with three major sets of public health education standards and competencies in mind, resulting in a few core learning objectives. The student objectives were to increase foundational knowledge and understanding of health determinants, practice systems thinking, engage in policy decision-making, and hone collaboration and leadership skills.

#### 2.3.1 Consortium of universities for global health competencies

The objective of enhancing foundational knowledge, as outlined in the Consortium of Universities for Global Health (CUGH) competencies (39), aims to help students gain an understanding of disease burden, social and environmental determinants of health, health inequities, cultural competency, and collaborative practices in public health.

#### 2.3.2 Council on education for public health

This pilot activity was also designed to incorporate several Council on Education for Public Health (CEPH) domains, foundational competencies, and cross-cutting concepts (37). The activity was intended to help students better understand the socioeconomic context in health policy through the analysis of economic factors such as tariffs and inflation, and how these factors affect health outcomes. Additionally, it was designed to provide exposure to the role of governance in managing public health initiatives, helping students become familiar with policy, governance, and advocacy domains and cross-cutting concepts. The activity was also designed to enhance students’ communication and teamwork skills through negotiation and alliance building, as well as to foster systems thinking by requiring students to integrate multiple components to address complex issues. Lastly, this activity met the domains and cross-cutting concepts for ethical and cultural sensitivity by demonstrating that promoting equitable and culturally respectful resource distribution is a challenging but critical endeavor in global health.

#### 2.3.3 Association of Schools and Programs of Public Health

The Association of Schools and Programs of Public Health (ASPPH) offers guidance for public health degree programs, emphasizing that graduates should have exposure to advanced policy analysis, leadership, and professional collaboration skills (72). This activity was designed to help students develop these skills by participating in policy negotiation and analysis, fostering leadership, teamwork, and handling complex scenarios.

## 3 Learning environment

The Global Health Trade Summit was designed for the Introduction to Global Health course at the University of Hawaiʻi at Mānoa. This course is facilitated twice a week in 75 min classes. This activity served as a midterm exercise within a series of Model United Nations activities (17). At the beginning of the semester, students were divided into 15 sovereign state teams, each representing a United Nations member state or the non-member observer state of Palestine.

The course had an enrollment of 39 undergraduate students in Fall 2024, and all attending students participated in the activity. Demographic characteristics for the study cohort were summarized using the program-level data because class-level demographics are not collected from any specific course for three reasons: (a) to protect privacy in a small group; (b) to minimize instructor burden; and (c) to comply with course and undergraduate education department committee policies. This approach avoids identifying students in a small class and reflects data privacy norms. Using program-level proxies leads to limitations: the researchers could not analyze outcomes by specific personal characteristics, which may limit the granularity of findings. We interpret results in aggregate. Introduction to Global Health is a core course taken early in the curriculum, so the age distribution of enrolled students may be slightly younger than the overall BAPH population. There is no evidence of differences by gender, race or ethnicity, residency, or other characteristics.

187 undergraduate students were enrolled in the BAPH degree program during the Fall 2024 semester. The mean age of students was 23 years old (SD = 5). More than 80% of students identified as female. Roughly two-thirds of students reported their permanent residence in Hawaiʻi. Almost all students reported their ethnicities. Almost half of students identified with multiple ethnicities. 44% identified as Caucasian or White; 34% identified as Filipino; 26% identified as Japanese; 24% identified as Chinese; and 18% identified as Native Hawaiian or Part-Native Hawaiian. About half of students indicated a career interest other than health, medicine, dentistry, pharmacy, law, or as a physician assistant. Mean GPA was 3.4 (SD = 0.6) out of 4, or a “B” grade.

### 3.1 Personnel and setting

#### 3.1.1 Personnel

In the activity, each team of two or three students assigned roles among themselves, choosing who would serve as trade representatives, health ministers, or finance ministers. Trade representatives left their stations to actively seek trading opportunities while health ministers remained at their stations to receive trade offers, and finance ministers assisted both.

Two educators facilitated this pilot activity: an instructor and a teaching assistant. The instructor represented the United States as the Trade Representative and facilitated trade negotiations as another participant in the simulation. The teaching assistant acted in a fictional role consolidated from roles at various U.S. agencies, including the USAID directorship, the Secretary of State, and representatives from the United States Congress—all involved in approving, providing, and distributing international aid. Simultaneously, the instructor and teaching assistant took advantage of teachable moments, shaped team discussion, and ensured that the activity met learning objectives. Together, they supported scenario development and execution.

#### 3.1.2 Setting

The physical and digital set-ups in the classroom were considered in activity design. There were designated spaces for each team to represent their respective sovereign states. These areas were arranged in the classroom in a manner that mimics a Western-standard world map; that is, sovereign states of a region were in close proximity. Due to the large number of students in a relatively small space, this arrangement allowed students to easily identify where other sovereign states may be located. Sovereign state name tent cards were also utilized to help students identify trading partners. The instructor and teaching assistant roamed the classroom during simulation rounds, but also claimed space in the corner of the classroom where North America sovereign states were located.

The pilot activity also provided options for seated participation to accommodate students with limited mobility. The instructor also established a policy allowing students to decline participation in the activity if they felt uncomfortable, upholding the program standard to provide a safe and comfortable learning environment for all students.

The digital set-up included a projector projecting onto two large screens in the front of the classroom, which displayed timers for each round. Also projected were Vox Media yearly recap videos (via YouTube) [e.g., (73)] and background music popular (via Apple Music) in different parts of the world relevant to the round of the activity. The digital set-up provided an additional sense of energy and content for students who wished to take a break from the highly active nature of the activity.

### 3.2 Learning objectives

This pilot activity was designed to promote seven key learning objectives aligned with global health education competencies as outlined above. The activity had students:

1. Articulate major global health challenges, including communicable disease spread, non-communicable disease burdens, environmental disasters, and sociopolitical upheavals.
2. Understand how social determinants and environmental factors influence health outcomes over time.
3. Analyze health systems, financing, governance, and trade policies across sovereign states.
4. Identify ethical considerations in population health interventions and uphold principles of social justice and equity.
5. Foster cultural humility and effective communication among sovereign states with diverse priorities and resources.
6. Gain a better understanding of epidemiological concepts and their appropriate usage in discussing global health matters.
7. Be better prepared to collaborate in multidisciplinary teams and demonstrate leadership through effective communication and advocacy.

All seven learning objectives are aligned with ASPPH’s emphasis on promoting the education of policy analysis, leadership, and professional collaboration skills. These skills are developed through students’ participation in complex multidisciplinary policy negotiation and analysis, which continue to foster leadership and teamwork.

### 3.3 Pedagogical format

#### 3.3.1 Development

**Figure 1:**
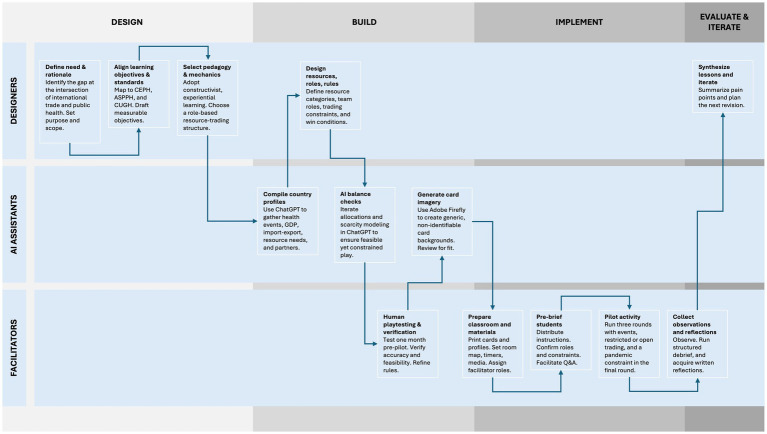
Flowchart of development and implementation process demonstrating role of AI assistance.

The activity development process is illustrated in Figure 1. Artificial intelligence (AI) tools played a role in the development of the Global Health Trade Summit activity, finding and packaging detailed, accurate, and engaging content. Specifically, OpenAI’s ChatGPT (Models GPT-3.5 Turbo & GPT-4), a generative AI chatbot (74), was extensively utilized to compile and analyze complex interdisciplinary data necessary for the simulation. This chatbot was used to gather historical information on each sovereign state, identifying significant events that majorly impacted population health. Additionally, ChatGPT assisted in constructing detailed economic profiles for each sovereign state, such as data on gross domestic product (GDP), related per capita economic indicators, and import–export portfolios. Moreover, this tool provided information about each sovereign state’s initial resource needs, changes in needs during the COVID-19 pandemic, and consequent changes to trade agreements or in trade relations.

Beyond data gathering, ChatGPT was instrumental in the design of the simulation activity itself. The chatbot identified and categorized the primary resources that each sovereign state would initially hold, as well as the resources they would need to obtain through trade. Also, ChatGPT was employed to run iterative simulations of the activity, verifying that resource allocations realistically met national needs through trade interactions, and verifying this could be replicated in the classroom. These test runs also ensured that the simulation realistically demonstrated resource scarcity, particularly in critical medical supplies during the pandemic year (2020). All calculations and scenario outcomes were done via ChatGPT, but their accuracy were subsequently verified through human-led playtesting 1 month prior to piloting the activity.

Another generative AI tool used in the development phase was Adobe Firefly (Version 3), a web-based application that makes images from textual descriptions (75). Firefly was specifically used to create visually appealing and contextual background imagery for each physical resource card, facilitating resource recognition. This approach provided visual aids for less familiar resources, such as cobalt or water treatment plants. Similarly, Firefly generated generic yet illustrative backgrounds for the event cards used during the activity. These images depicted potential scenarios without portraying specific populations or identifiable geographic locations, thereby protecting privacy and preventing misrepresentation. Event card images illustrated general resources: for example, using visuals of nondescript aid rations or agricultural goods on cards representing disruptions caused by natural disasters affecting food supplies. Each AI-generated image was reviewed by the instructor to ensure visual accuracy, relevance to the respective resource or event, and ethical compliance.

#### 3.3.2 Facilitation

The facilitation of the Global Health Trade Summit began in the preceding class session, where the final 15 min were dedicated to preparing students for the activity. During this time, students were provided with detailed instructions (Appendix A), which they reviewed individually and as a pre-established group associated with previously initiated Model UN activities. The instructions outlined the mechanics of the activity, including their assigned roles (Trade Representative, Health Minister, and Finance Minister, if applicable), trading restrictions, and the importance of collaboration and strategy. Students were also introduced to their sovereign state profiles (Appendix B), which included their starting resources, key needs, and close trading partners, along with a brief overview of their population’s health issues for the years 2018 and 2019. This preparatory step allowed students to familiarize themselves with the materials and develop initial trading strategies and priorities with their team members.

Groups received 3–10 resource cards, depending on the GDP of the sovereign state they represented. These resources were organized into categories: food supplies (e.g., fruits, soybeans, spices); medical supplies (e.g., pharmaceuticals, vaccines); economic goods (e.g., oil, textiles, cobalt); and clean water (e.g., conservation systems, water treatment plants).

The activity itself was conducted during the last hour of a 75 min class session. The first 15 min of the class were dedicated to setup, unrelated announcements, and global news. The activity consisted of three roughly 10 min rounds, representing the years 2018, 2019, and 2020. In total, the Global Health Trade Summit took a total of 75 min: an hour for the activity and 15 min for instruction delivery during the preceding class.

**Figure 2:**
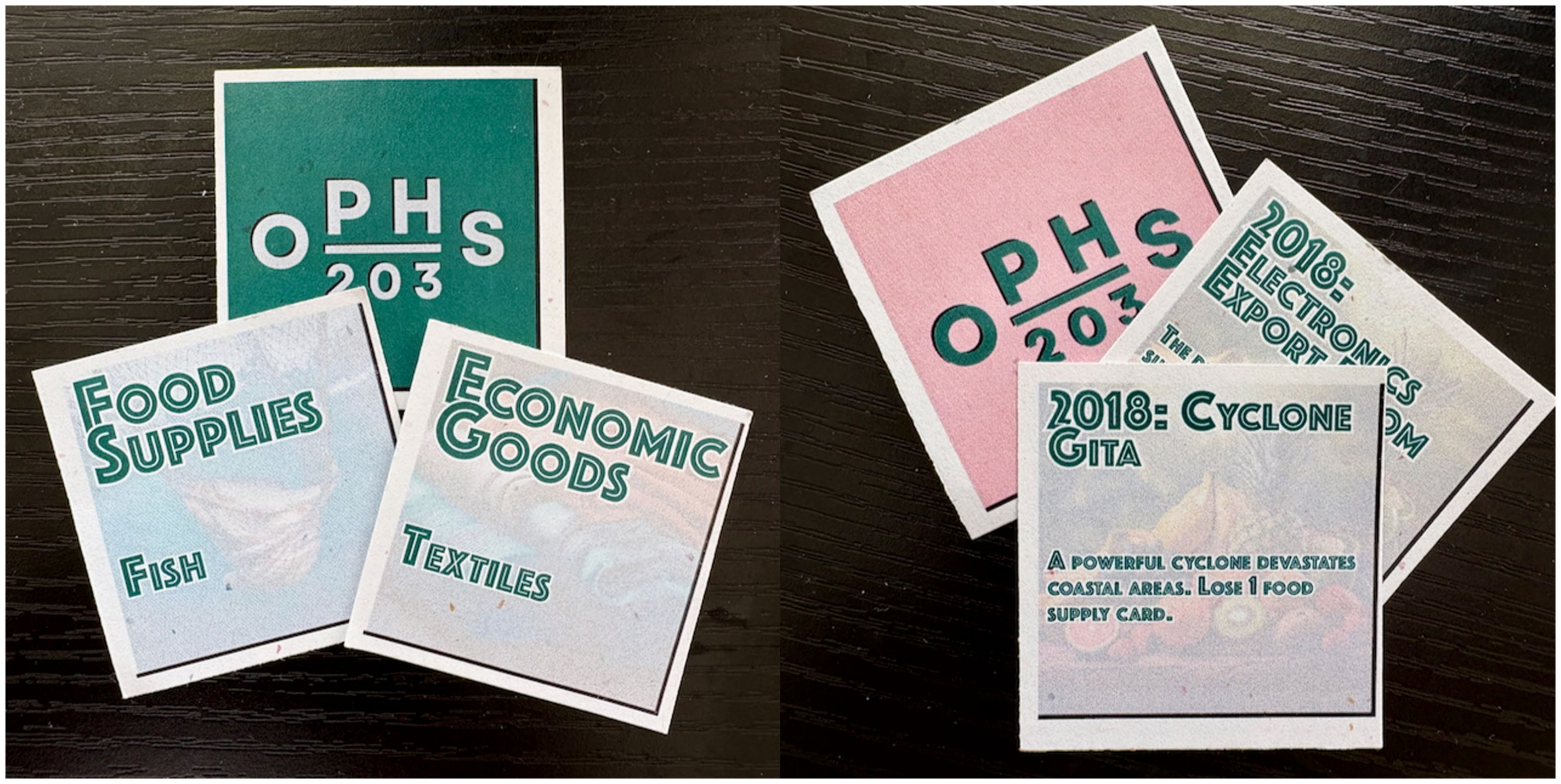
Graphics of sample resource cards and event cards used in simulation.

Each round began with students reviewing their available resources and needs, as specified in their sovereign state profiles. For most of each round, students were restricted to trading with their close trading partners, a limitation that was relaxed during the final minutes of each round to allow broader trade opportunities. During these periods, Trade Representatives moved around the classroom, negotiating trades with or offering aid to other sovereign states, while Health Ministers managed incoming trades and offers from their team’s designated area. If a team included a Finance Minister, that student tracked resources and provided strategic advice. Event cards (Appendix C), representing real-world disruptions or opportunities, were introduced in each round by the instructor. These cards altered the trading landscape by adding unexpected challenges or opportunities, such as resource gains or losses, simulating global trade complexities. Examples include generic events (e.g., foreign aid influx, natural disaster, economic boom, health crisis, trade embargo) and nation state-specific events (e.g., mineral discovery in the Democratic Republic of Congo, cholera resurgence in Haiti). Examples of resource and event cards can be seen in Figure 2.

The final round, representing the year 2020, introduced significant restrictions to mirror the impact of the COVID-19 pandemic. Most teams were tasked with securing critical resources, such as personal protective equipment, vaccines, and pharmaceuticals, while facing limited trading options due to simulated lockdowns, but some teams were asked to consider providing aid to regional allies or underserved sovereign states. As each round concluded, students reflected briefly on their transactions, discussing how their strategies impacted their sovereign state’s health and economy. These reflections culminated in a larger debriefing session at the end of class and continuing into the next class, where students analyzed outcomes by region and globally, highlighting key lessons on trade, health disparities, and resource allocation.

## 4 Results

### 4.1 Processes and tools

The Global Health Trade Summit utilized a combination of observational techniques and informal evaluation of written reflections to collect aggregate data on student engagement, strategies, and decision-making processes. The data reported were approved by the University of Hawaiʻi Institutional Review Board (Protocol #2025-00584). Data were gathered through three complementary sources: real-time observations, facilitated discussions, and written reflections. In accordance with extant course policies, activity components, such as group discussions and debriefings, were not captured by audio or video. This approach was intended to safeguard student privacy and foster open and authentic dialogue, particularly in a setting where many peers were interacting for the first time.

During the simulation, the instructor and teaching assistant closely monitored student interactions, focusing on the negotiations led by Trade Representatives, the management of resources by Health Ministers, and the advisory roles of Finance Ministers. The instructor and teaching assistant closely observed interactions using an unstructured observation guide. This guide focused on behaviors (e.g., key decisions, rationales, questions raised) tied to our learning objectives; for example, how each team negotiated trades, managed resources, and coordinated roles. Observations were documented in real-time, particularly during the reflective periods between rounds, when students regrouped to discuss strategies and evaluate their performance. This provided the teaching team with valuable insights into group dynamics and decision-making processes.

The teaching team also leveraged the mechanics of the activity itself to gather insights. The use of event cards, trading restrictions, and evolving resource needs allowed for the observation of how students adapted to changes in the simulation environment. The structure of the rounds—starting with restricted trading and concluding with open trading and aid issuance—was designed to highlight shifts in behavior and collaboration patterns.

The structured post-activity debriefing session was another critical tool for gathering data. This session followed a tiered approach: students first reflected within their sovereign state groups, then discussed outcomes by regional blocs, and finally participated in a full-class discussion. The structured format allowed the teaching team to capture a range of perspectives, from individual group strategies to broader observations about global trade and health systems.

In addition to the debrief, students were assigned a take-home written reflection to be submitted within 48 h after the activity (Appendix D). These reflections encouraged students to articulate their individual experiences in detail, providing informal qualitative data on their understanding of the activity’s challenges and implications. These reflection responses were reviewed by the teaching team to supplement the observational data.

After the session, the instructors compared notes and reconciled any discrepancies to ensure interrater reliability through a consistent interpretation of events. This method of dual-observer note-taking and cross-checking helped enhance the reliability of our qualitative data. We then aggregated the observation notes to identify common themes and notable incidents. Notably, each note was coded to reflect evidence of the learning objectives.

By combining direct observation, structured discussions, and individual written reflections, the activity provided a comprehensive framework for collecting data on student engagement and decision-making processes. Due to the nature of this pilot activity, more formal analytic techniques were not used to detect themes or patterns.

### 4.2 Data gathered

The data gathered from the Global Health Trade Summit revealed insights into student behavior, learning, and engagement. Observations by the teaching team during the simulation showed that students quickly adapted to their roles, with Trade Representatives actively negotiating trades and aid packages, and Health Ministers managing incoming offers. Students reported in activity debriefings that teams with Finance Ministers allowed more deliberate planning and resource management, and self-reported achieving more strategic outcomes and more aid delivery compared to teams without this role. Throughout the simulation, students refined their strategies, initially focusing on immediate needs and close trading partners, but eventually broadening their approach to include alliances and resource-sharing agreements with a wider range of sovereign states.

Debriefing also showed that the final round, which simulated the global trade disruptions caused by the COVID-19 pandemic, prompted a notable shift in student behavior. With stricter trading constraints, students were forced to prioritize critical resources like PPE and vaccines, leading to intense negotiations and creative problem-solving. Many students noted that this round was particularly impactful in illustrating the real-world challenges of addressing health needs during a crisis. Event cards also played a key role in influencing student strategies, as unexpected gains or losses of resources forced teams to reassess their priorities and adapt their plans in real time.

The post-activity debrief written reflections further highlighted the depth of student learning. Most students reported an increased appreciation for the complexity of global trade and the ethical challenges of resource allocation. Many recognized inequities faced by resource-limited sovereign states and expressed a deeper understanding of systems thinking, acknowledging how trade, aid, health outcomes, and economic factors are interconnected. The activity also revealed how students grappled with ethical dilemmas, such as deciding which populations to prioritize when resources were scarce.

For the instructors, the data supported this pilot activity’s effectiveness in fostering critical thinking, collaboration, and an appreciation for global health complexities. The combination of real-time observations, structured debrief, and individual reflections provided a rich understanding of how the simulation engaged students and met its educational objectives.

## 5 Discussion

### 5.1 Activity reflections

The implementation of this activity significantly enhanced students’ foundational knowledge, understanding of health determinants, and ethical considerations in the health field. Additionally, it provided students with practical experience in systems thinking, and fostered collaboration and leadership skills through dynamic simulation. Furthermore, the activity aligned with the competencies and standards required for an undergraduate public health course, ensuring wide coverage of essential principles.

Many students demonstrated basic understanding of how international trade policies and economic factors, such as tariffs and supply chain disruptions, can impact population health. Additionally, they gained a more profound comprehension of the multifaceted impact of the COVID-19 pandemic on population health, extending beyond the evident transmission of communicable diseases and the consequential health outcomes associated with them. They also grappled with ethical decision-making and cultural humility, often discussing strategies for equitable resource distribution. Some students mentioned that the pilot activity helped them understand the need and provision of aid during the ongoing conflict in Palestine. Students honed their abilities of alliance building and negotiation—essential to address real-world public health challenges, particularly when evaluating or formulating population health policies.

The results of the Global Health Trade Summit highlight several lessons that resonate with research on experiential and game-based learning. First, by allowing students to learn-by-doing in a risk-free environment, this gamified simulation enabled them to apply concepts and refine skills without real-world repercussions, which is known to foster confidence, teamwork, and critical thinking (76, 77).

In this context, students engaged in experimenting with international policy decisions and observing their outcomes, which rendered abstract concepts tangible. The enthusiastic participation and complexity and riskiness of strategies that emerged are consistent with the engagement observed in other gamified public health exercises, such as outbreak response simulations and health systems role-plays (78, 79). Students sustained attention throughout multiple rounds and exhibited the ability to recall trade dynamics and health implications subsequently. This aligns with research in health professions education demonstrating that gamification can substantially enhance learning outcomes and student motivation (79, 80).

Our observations of students collaborating under pressure and systematically considering resource distribution corroborate findings of researchers who have identified that gamified learning environments foster active learning and critical thinking by simulating real-world challenges in a structured format (76, 80).

The experiential learning cycle was pivotal. This supports the widely recognized importance of debriefing for reinforcing learning concepts and facilitating metacognitive processing (81–83). By comparing strategies and outcomes during the debrief, students engaged in the reflective observation and abstract conceptualization advocated by Kolb’s experiential learning model (20).

Literature on public health simulations similarly emphasizes guided reflection as a crucial factor in translating experience into enduring knowledge acquisition (20, 84, 85). In our activity, these discussions enabled students to connect the simulation to broader public health themes, such as equity and policy trade-offs, thereby reinforcing these connections in their memory. This type of experiential learning is precisely what contemporary public health education models advocate for, combining knowledge with practical skills and civic perspective (37, 72).

The Global Health Trade Summit successfully addressed all learning objectives, either fully or partially:

- Objectives 1 & 6: Students’ foundational knowledge of global health trade improved and in post-activity reflections many correctly articulated how tariffs, supply chain disruptions, or aid policies affect population health in the real world. Students were observed utilizing epidemiological concepts and sharing health indicators when giving rationale for proposed trades in the activity and also in follow-up reflections.
- Objective 2: The objective of understanding health determinants and disparities was met as well; during discussions, students frequently identified how poorer countries in the simulation suffered worse outcomes over a lifetime (and generations), echoing real health inequities.
- Objectives 3 & 5: We also saw engagement with policy decision-making when students tackled trade-offs during the COVID-19 crisis round, such as deciding whether to impose export bans or to provide foreign aid despite domestic needs. Several groups explicitly debated these policy dilemmas while providing context of their (and other) countries’ health systems and needs, mirroring real-world governance decisions. Groups also provided culturally grounded reasons for decisions.
- Objective 4: Additionally, students grappled with ethical considerations in resource allocation, as witnessed by the instructors extensively during the debrief. Many wrote about the difficulty of deciding who to help initially, and they reflected on principles of equity and justice, indicating growth in ethical reasoning.
- Objective 7: The development of collaboration and leadership skills was apparent throughout the simulation, and teams that communicated effectively seemed to trade more frequently and were pleased with their trades. The learning goal of applying systems thinking was evident as teams formed strategic alliances and integrated economic and health considerations in their trading practices.

In summary, the behaviors we observed mapped back to the intended learning objectives, confirming that the activity achieved its educational aims.

### 5.2 Practical implications

Positive student outcomes and increased engagement were achieved through the interdisciplinary nature of the exercise. Students gained a holistic understanding of global health issues by integrating cultural, economic, and philosophical concepts into their broader base of public health and global health knowledge. These interdisciplinary insights enable students to address macro-level questions about the complexity of public health issues and policy solutions. Also, by utilizing their knowledge and skills in other courses and fields of study, students bridge the gap between their previous and new knowledge, allowing for new insights and perspectives. Another practical implication is this activity design allows it to be implemented in larger classes and in different physical environments with little to no modification. It is suitable for use in other degree programs aligned with key public health education standards.

In essence, this simulation demonstrates the potential of generative AI tools in higher education. The combination of ChatGPT for development and playtesting, and Adobe Firefly for image generation, proved highly effective for making activities, allowing the instructors to envision the activity in advance and produce visually engaging materials that deepened immersion. The implications of employing generative AI tools to develop learning activities are significant: for example, instructors can rapidly prototype multiple scenario variations and tailor content to different grade levels. Instructors can even automate the integration of real-time data into their curricula. Similarly, generative AI tools can easily create educational graphics, like backdrops for resource cards illustrating rare materials or region-specific events, leading to greater accessibility and engagement. The widespread adoption of such tools can lead to the creation of more creative and intricate simulations among groups. This innovation holds the potential to usher in a golden age of activity and curriculum design.

Although incorporating generative AI in the development of this activity offers numerous benefits, it is essential to acknowledge the inherent limitations, risks, and ethical considerations associated with educational AI tools. One primary concern pertains to the accuracy and reliability of AI-generated content, including introducing errors or hallucinations that could distort the learning experience, as highlighted in the landmark UNESCO report on safe and fair usage of generative AI in education and subsequent literature reviews (86–88). In this context, all country-specific data and scenario outcomes provided by ChatGPT underwent cross-checking against authoritative sources before playtesting to guarantee accuracy. Additionally, the instructors maintained monitored output for bias. AI systems are trained on extensive datasets that may harbor cultural biases (86, 89, 90), which can inadvertently manifest in the historical information presented on cards.

To address this, the cards were meticulously reviewed for any biased or stereotypical portrayals of countries. None were found, but the instructors had planned to make necessary adjustments to ensure cultural fairness and respect. Similarly, all AI-generated images were subjected to a screening process to ascertain their appropriateness. For instance, we avoided any visuals that could inadvertently stigmatize or single out specific real people or communities, opting instead for abstracted illustrations or commonplace graphics.

The instructors recognize the imperative of human oversight when employing generative AI in educational settings. While AI served as a valuable tool in the design of the simulation, it did not supplant the instructors’ judgment. To maintain transparency with students, we explicitly communicated AI’s role, explaining that certain background data and graphics were AI-assisted, multiple times, including when students commented on the imagery on the cards during trading rounds. This transparency was necessary to prevent any misconception of AI as an infallible authority. In accordance with best practices, our implementation treated AI output as preliminary drafts subject to expert review, rather than presenting them as definitive truths.

The authors have considered educational equity and integrity concerns. Specifically, not all students have access to AI tools, possess AI literacy to utilize such tools, or understand the ethical considerations of utilization of or over-reliance on AI for assignments. Although in this simulation, AI was utilized by instructors (and not directly by students), the lead instructor used this learning opportunity to comment on the appropriate applications and limitations of AI. In summary, the incorporation of generative AI necessitated meticulous ethical safeguards: verifying the accuracy of content, ensuring cultural sensitivity, maintaining human oversight, and educating students about the appropriate role of AI in the learning process.

### 5.3 Lessons learned

Several valuable lessons were gained during the development and implementation of the pilot activity. Four notable lessons stand out.

First, insufficient time was allocated for student support due to the instructor and teaching assistant’s multiple responsibilities during the activity. This not only exacerbated any confusion students might have had about gameplay but also prevented teaching moments, which are a hallmark of this activity. In the future, the role of the instructor may be better served in a supportive, neutral observer role than as a specific sovereign state, or additional instructional support may be needed for the activity day.

Second, students struggled to adhere to numerous game rules. The instructor faced challenges in clearly explaining the instructions. The instructor had to repeatedly interrupt gameplay to introduce rules that should have been made clearer at the beginning. Giving instructions took up a larger portion of the activity’s total time compared to other activities. Countless minor changes to the gameplay, mechanics, rules, or objectives could have streamlined the simulation, recentering student focus on learning content rather than mastering the game. In future iterations, instructors should practice giving full instructions in advance and create more opportunities for students to inquire into the game’s objectives, rules, and gameplay mechanics before the simulation begins.

Third, extended discussion and reflection time are crucial to reinforce the key learnings from the activity. Integrating additional discussion and reflection within the activity and between rounds would have provided students with chances to grasp and plan their actions. Allowing students dedicated reflection time would enable them to self-assess their academic outcomes (i.e., game performance) more comprehensively and in real time, rather than retrospectively.

Fourth, significant learning took place in the field of AI-driven tools since the inception of this simulation activity, particularly in the area of prompt engineering and its application in designing educational activities. As technology advances, instructors may find it easier to create these activities using these tools, with greater ease and intuition.

## 6 Constraints and limitations

This pilot activity was conceived solely as an educational activity and was not intended as formal research involving human subjects. This approach simplified planning, development, and implementation but restricted the scope and quality of data collected on academic, behavioral, and affective outcomes.

This activity necessitates multiple facilitators and ample space for the simulation to progress at a pace that is fast enough to justify valuable class time and slow enough to address student questions and provide opportunities for ad-hoc instruction.

Utilizing artificial intelligence tools inherently introduces misinformation and unintentional biases. Therefore, a meticulous review of the learning content is crucial, even as generative AI tools yield more reliable and valid information.

This activity was designed in the post-pandemic era, where residual complexities from pandemic-era education must be taken into account (24, 91). There is significant variation in students’ abilities in those who attended high school online, and multi-part live activities may not be familiar to many students.

There are numerous physical resources in this game, which require basic printing facilities. Using digital resources would be an alternative, but it introduces new requirements (and distractions) for students, while instructors must utilize a tracking system and be prepared to troubleshoot issues.

Overall, constraints were minor, and student gains were meaningful. The Global Health Trade Summit is just one example of many engaging interdisciplinary activities for undergraduate public health students that can be developed easily with widely available AI tools.

## Data Availability

The raw data supporting the conclusions of this article will be made available by the authors, without undue reservation.
