# Supplementary material for: Global Health Trade Summit: an AI-enhanced simulation of international trade and global health for undergraduate public health education
Source: Front Public Health. 2025 Nov 14;13:1681199. doi: 10.3389/fpubh.2025.1681199 (PMC12660194; doi:10.3389/fpubh.2025.1681199)
Supplement: Supplementary file 1 [file Table_1.docx]

Supplementary Material

# Appendix A. Instructions

| **Objective:** Explore how trade with other sovereign states can impact your nation’s health and economy.  **Instructions**   - **Represent your assigned sovereign state** during the trade summit, focusing on health and resources. - **Review your sovereign state profile**. It shows your resources, health issues, and trading partners. You can only trade with the sovereign states listed as your partners. - **Assign roles** within your team: one person as the Trade Representative (they go out to other sovereign states to negotiate trades and aid packages), one as the Health Minister (they stay at home to manage trades and accept incoming offers), and, if you have a third person, one as the Finance Minister (they track resources and provide strategic advice). - **Trade** during each round**.** The Trade Representative will negotiate trades with other sovereign states, while the Health Minister manages trades from your base, accepting and negotiating offers from foreign Trade Representatives. The Finance Minister, if you have one, will give advice and monitor the sovereign state’s resources. You may give resources away as part of aid packages. - **Continue for three rounds**, representing the years 2018, 2019, and 2020. - **Use your resources** to trade with your partners to meet your key needs and improve health. - **Be prepared** as event cards will be drawn throughout the game and may disrupt your trade by introducing new challenges or opportunities. Adjust your strategy as needed to respond to these events. - **Reflect** on how your trades affected your sovereign state’s health and economy after each trading phase. - **Debrief** at the end of the game to discuss which strategies worked best and how trade impacted global health. |
| --- |

# Appendix B. Sovereign State Profiles and Resource Cards

| **Afghanistan**   - Health Issues (2018): Malnutrition, Tuberculosis - Health Issues (2019): Malnutrition, Infectious Diseases - Starting Resources: Food Supplies (Fruits), Clean Water (Mountain Springs), Economic Goods (Textiles) - Key Needs: Medical Supplies, Clean Water, Economic Goods - Trading Partners: India, Pakistan, Yemen   **Brazil**   - Health Issues (2018): Obesity, Cardiovascular Diseases - Health Issues (2019): Zika Virus, Non-communicable Diseases - Starting Resources: Food Supplies (Soybeans), Food Supplies (Coffee), Medical Supplies (Pharmaceuticals), Economic Goods (Textiles), Economic Goods (Sugarcane), Clean Water (Water Treatment Plants) - Key Needs: Clean Water, Medical Supplies, Economic Goods - Trading Partners: United States, Colombia, Mexico   **Colombia**   - Health Issues (2018): Malnutrition, Dengue Fever - Health Issues (2019): Malaria, Non-communicable Diseases - Starting Resources: Food Supplies (Bananas), Food Supplies (Coffee), Clean Water (Natural Springs), Economic Goods (Textiles) - Key Needs: Medical Supplies, Economic Goods, Clean Water - Trading Partners: United States, Brazil, Mexico   **Democratic Republic of Congo**   - Health Issues (2018): Malnutrition, Malaria - Health Issues (2019): Ebola Virus, HIV/AIDS - Starting Resources: Economic Goods (Cobalt), Food Supplies (Coffee), Clean Water (Rainforest Sources) - Key Needs: Medical Supplies, Food Supplies, Clean Water - Trading Partners: Kenya, India, Nigeria   **Fiji**   - Health Issues (2018): Non-communicable Diseases, Waterborne Diseases - Health Issues (2019): Tropical Diseases, Diabetes - Starting Resources: Food Supplies (Fish), Clean Water (Artesian Water), Economic Goods (Coconut Oil) - Key Needs: Medical Supplies, Food Supplies, Clean Water - Trading Partners: Philippines, India, United States   **Haiti**   - Health Issues (2018): Malnutrition, Cholera - Health Issues (2019): Tuberculosis, Dengue Fever - Starting Resources: Food Supplies (Coffee), Clean Water (Spring Water), Economic Goods (Handicrafts) - Key Needs: Medical Supplies, Clean Water, Economic Goods - Trading Partners: United States, Mexico, Brazil   **India**   - Health Issues (2018): Malnutrition, Tuberculosis - Health Issues (2019): Air Pollution, Dengue Fever - Starting Resources: Medical Supplies (Pharmaceuticals), Medical Supplies (Vaccines), Food Supplies (Rice), Food Supplies (Spices), Economic Goods (Textiles), Clean Water (Conservation Systems), Economic Goods (Automobiles) - Key Needs: Clean Water, Food Supplies, Medical Supplies - Trading Partners: Afghanistan, Kenya, Philippines   **Kenya**   - Health Issues (2018): Malaria, HIV/AIDS - Health Issues (2019): Malnutrition, Non-communicable Diseases - Starting Resources: Food Supplies (Tea), Food Supplies (Fruits), Clean Water (Lake Sources), Economic Goods (Flowers) - Key Needs: Medical Supplies, Clean Water, Economic Goods - Trading Partners: India, Democratic Republic of Congo, Nigeria   **Mexico**   - Health Issues (2018): Diabetes, Obesity - Health Issues (2019): Non-communicable Diseases, Respiratory Infections - Starting Resources: Food Supplies (Avocados), Food Supplies (Corn), Medical Supplies (Medical Devices), Economic Goods (Oil), Economic Goods (Electronics), Clean Water (Treatment Facilities) - Key Needs: Clean Water, Medical Supplies, Economic Goods - Trading Partners: United States, Colombia, Haiti   **Nigeria**   - Health Issues (2018): Malaria, HIV/AIDS - Health Issues (2019): Cholera, Non-communicable Diseases - Starting Resources: Economic Goods (Oil), Food Supplies (Cocoa), Food Supplies (Yams), Clean Water (River Sources), Medical Supplies (Herbal Remedies) - Key Needs: Clean Water, Food Supplies, Medical Supplies - Trading Partners: Kenya, India, Democratic Republic of Congo   **Palestine**   - Health Issues (2018): Waterborne Diseases, Malnutrition - Health Issues (2019): Respiratory Infections, Diabetes - Starting Resources: Food Supplies (Olives), Clean Water (Spring Sources), Economic Goods (Textiles) - Key Needs: Clean Water, Medical Supplies, Food Supplies - Trading Partners: Yemen, India, Afghanistan   **Philippines**   - Health Issues (2018): Dengue Fever, Tuberculosis - Health Issues (2019): Measles, Non-communicable Diseases - Starting Resources: Food Supplies (Rice), Food Supplies (Coconuts), Economic Goods (Electronics), Clean Water (Mountain Springs), Economic Goods (Support Services) - Key Needs: Medical Supplies, Clean Water, Food Supplies - Trading Partners: United States, India, Fiji   **Republic of the Marshall Islands**   - Health Issues (2018): Non-communicable Diseases, Waterborne Diseases - Health Issues (2019): Diabetes, Tropical Diseases - Starting Resources: Food Supplies (Fish), Clean Water (Conservation Systems), Economic Goods (Shipping Services) - Key Needs: Medical Supplies, Food Supplies, Clean Water - Trading Partners: Philippines, United States, Fiji   **United States**   - Health Issues (2018): Obesity, Heart Disease - Health Issues (2019): Opioid Crisis, Respiratory Infections - Starting Resources: Medical Supplies (Vaccines), Medical Supplies (Pharmaceuticals), Food Supplies (Grains), Food Supplies (Meat), Food Supplies (Livestock), Economic Goods (Technology), Economic Goods (Automobiles), Economic Goods (Machinery), Clean Water (Purification Technology), Clean Water (Bottled Water) - Key Needs: Clean Water, Economic Goods, Food Supplies - Trading Partners: Mexico, Brazil, Philippines   **Yemen**   - Health Issues (2018): Malnutrition, Cholera - Health Issues (2019): Tuberculosis, Dengue Fever - Starting Resources: Food Supplies (Coffee), Clean Water (Aquifers), Economic Goods (Textiles) - Key Needs: Medical Supplies, Clean Water, Food Supplies - Trading Partners: Palestine, Afghanistan, Philippines |
| --- |

# Appendix C. Event Cards

| **Events**  *Generic*   - Natural Disaster: A hurricane or earthquake hits one of your trading partners, disrupting trade. You cannot trade with this partner during the current round. - Economic Boom: Your sovereign state experiences a surge in economic growth. Gain 2 additional resource cards. - Health Crisis: A sudden health crisis emerges in your sovereign state. You must use a medical supplies card to address the situation before making any other trades. - Trade Embargo: A major trading partner imposes an embargo on one of your key exports. Choose one resource card that you cannot trade this round. - Foreign Aid: An international organization provides an aid package to your sovereign state. Gain 1 clean water card and 1 food supply card.   *Specific*  Afghanistan   - Drought (2018): Severe droughts across the sovereign state reduce agricultural output. Lose 1 food supply card. - Kabul Security Crisis (2019): A major attack disrupts trade routes and creates instability. You cannot trade with any sovereign state for this round. - Wheat Harvest Boom (2018): Favorable weather conditions result in a bumper wheat harvest. Gain 1 food supply card.   Brazil   - Truck Drivers’ Strike (2018): A national truck drivers’ strike paralyzes transportation and affects the supply chain. Lose 1 economic goods card. - Amazon Fires (2019): Massive fires in the Amazon forest disrupt agriculture. Lose 1 food supply card. - EU Trade Agreement (2019): Brazil signs a new trade agreement with the European Union, boosting exports. Gain 1 economic goods card.   Colombia   - Venezuelan Refugee Crisis (2018): An influx of refugees puts pressure on your sovereign state’s resources. Lose 1 clean water card. - Cocaine Trade Crackdown (2019): Increased enforcement disrupts economic goods exports. You cannot trade economic goods this round. - Coffee Market Expansion (2018): Increased demand for Colombian coffee in international markets. Gain 1 food supply card.   Democratic Republic of Congo   - Ebola Outbreak (2018): An Ebola outbreak overwhelms health systems. You must use a medical supplies card to contain the outbreak before making any trades. - Mining Protests (2019): Protests in mining regions disrupt economic goods production. Lose 1 economic goods card. - Mineral Discovery (2019): A new cobalt mine is discovered, boosting the sovereign state’s economy. Gain 1 economic goods card.   Fiji   - Cyclone Gita (2018): A powerful cyclone devastates coastal areas. Lose 1 food supply card. - Water Shortage (2019): A prolonged drought causes clean water scarcity. You cannot trade clean water this round. - Tourism Boost (2018): A surge in tourism provides extra funds for the economy. Gain 1 economic goods card.   Haiti   - Earthquake Aftershocks (2018): Aftershocks from the 2010 earthquake continue to damage infrastructure. Lose 1 economic goods card. - Cholera Resurgence (2019): A cholera outbreak resurfaces. You must use a clean water card to address the crisis before making any trades. - International Aid Package (2019): A major international aid organization provides essential supplies. Gain 1 clean water card and 1 medical supplies card.   India   - Air Pollution Crisis (2018): Severe air pollution disrupts public health. You must use a medical supplies card to mitigate the health crisis before making any trades. - Flooding in Kerala (2019): Massive floods damage agriculture in Kerala. Lose 1 food supply card. - Record Rice Production (2018): Ideal weather conditions lead to a record rice harvest. Gain 1 food supply card.   Kenya   - Drought in the Horn of Africa (2018): Severe drought affects food production. Lose 1 food supply card. - Locust Invasion (2019): A locust invasion destroys crops. You cannot trade food supplies this round. - Tea Export Success (2019): Kenya’s tea exports hit a new high due to increased global demand. Gain 1 economic goods card.   Marshall Islands   - Rising Sea Levels (2018): Rising sea levels damage freshwater sources. Lose 1 clean water card. - Nuclear Waste Leakage (2019): Leakage from a nuclear waste site contaminates coastal waters. You must use a clean water card to address the issue before making any trades. - Infrastructure Upgrade (2018): Funding from international partners allows for the construction of new rainwater collection systems. Gain 1 clean water card.   Mexico   - Hurricane Willa (2018): A hurricane hits the Pacific coast, damaging agricultural regions. Lose 1 food supply card. - Fuel Shortage (2019): A fuel pipeline theft crisis leads to a national fuel shortage. Lose 1 economic goods card. - US Trade Deal (2019): A favorable trade agreement with the United States boosts the economy. Gain 1 economic goods card.   Nigeria   - Boko Haram Attack (2018): An attack disrupts trade in the northern region. You cannot trade with any sovereign state for this round. - Flooding (2019): Heavy floods damage agricultural lands. Lose 1 food supply card. - Agricultural Innovation (2018): New agricultural techniques result in a bumper crop of yams. Gain 1 food supply card.   Palestine   - Gaza Electricity Crisis (2018): Electricity shortages limit water treatment. Lose 1 clean water card. - Border Closure (2019): Political tensions lead to border closures. You cannot trade with any sovereign state for this round. - Olive Harvest Success (2019): An exceptional olive harvest provides economic stability. Gain 1 food supply card.   Philippines   - Typhoon Mangkhut (2018): A powerful typhoon damages crops and infrastructure. Lose 1 food supply card. - Measles Outbreak (2019): A measles outbreak affects public health. You must use a medical supplies card to contain the outbreak before making any trades. - Electronics Export Boom (2018): The demand for electronics surges, increasing national income. Gain 1 economic goods card.   United States   - California Wildfires (2018): Wildfires devastate agricultural regions. Lose 1 food supply card. - Government Shutdown (2019): A federal government shutdown disrupts trade. You cannot make any trades this round. - Medical Research Breakthrough (2019): A new vaccine development provides a boost to the medical industry. Gain 1 medical supplies card.   Yemen   - Civil War Escalation (2018): The ongoing civil war intensifies, disrupting trade. You cannot trade with any sovereign state for this round. - Cholera Outbreak (2019): A cholera outbreak overwhelms the healthcare system. You must use a clean water card to address the crisis before making any trades. - Humanitarian Mission (2018): A successful humanitarian mission provides essential supplies. Gain 1 clean water card and 1 food supply card. |
| --- |

## Appendix D. Written Reflection

| **Please reflect on your experience during the activity and answer the following questions:**  Name: _______________________________________  Member State: _________________________________  Which sovereign states did you trade with the most?  What types of resources did your sovereign state import (or receive as aid) the most?  What types of resources did your sovereign state export the most?  What challenges or difficulties did you encounter during the trading process?  How did the event cards affect your sovereign state’s ability to trade?  Was it easier or harder for your sovereign state to complete the activity compared to others? |
| --- |
